# The Medical News Visiting List for 1891

**Published:** 1891-01

**Authors:** 


THE MEDICAL NEWS VISITING LIST FOR 1891. Philadelphia. Lea Brothers & Co.

Having been thoroughly revised, their visiting list is quite up to date. The table of contents is full, and we note that there is much useful data contained within the handsome red leather covers. Artificial respiration, incompatibles, poisons and antidotes, ligation of arteries, &c., are sufficiently touched upon, to make this little book quite sufficient for reference in emergencies. The blanks are classified and arranged in visits to patients. Records of all kinds of professional work with memoranda and accounts. In its totality, we deem it quite superior, and decidedly capable of fulfilling the purpose for which it is intended.

